# Decreased Temperature Sensitivity of *Vestigial* Gene Expression in Temperate Populations of *Drosophila melanogaster*

**DOI:** 10.3390/genes10070498

**Published:** 2019-06-28

**Authors:** Susanne Voigt, Anna Christina Erpf, Wolfgang Stephan

**Affiliations:** 1 Section of Evolutionary Biology, Department of Biology II, University of Munich, Planegg-Martinsried, 82152 Munich, Germany; 2 Applied Zoology, Faculty of Biology, Technische Universität Dresden, 01217 Dresden, Germany; 3 Lunenfeld-Tanenbaum Research Institute, Mount Sinai Hospital, Toronto, ON M5G 1X5, Canada; 4 Leibniz Institute for Evolution and Biodiversity Science, 10115 Berlin, Germany

**Keywords:** evolution of gene regulation, environmental sensitivity, expression plasticity, temperature-dependent expression

## Abstract

*Drosophila melanogaster* recently spread from its tropical origin in Africa and became a cosmopolitan species that has adapted to a wide range of different thermal environments, including temperate climates. An important limiting factor of temperate climates has probably been their low and varying temperatures. The transcriptional output of genes can vary across temperatures, which might have been detrimental while settling in temperate environments. The reduction of temperature-sensitive expression of functionally important genes to ensure consistent levels of gene expression might have been relevant while adapting to such environments. In this study, we focus on the gene vestigial (*vg*) whose product is a key factor in wing development. We provide evidence that temperature-sensitivity of *vg* has been buffered in populations from temperate climates. We investigated temperature-sensitivity of *vg* gene expression in six natural populations, including four temperate populations (three from Europe and one from high-altitude Africa), and two tropical populations from the ancestral species range. All temperate populations exhibited a lower degree of temperature-induced expression plasticity than the tropical populations.

## 1. Introduction

Species colonizing new environments need to adapt to novel biotic and abiotic conditions. One critical determinant of species ranges that varies with latitudes and altitudes is environmental temperature. In particular for ectotherms, such as insects, temperature plays a major role in determining species abundance and geographic distribution [1,2]. As a cosmopolitan species, the fruit fly *Drosophila melanogaster* has adapted to a wide range of thermal environments [3,4,5]. Its origin is thought to be in tropical southern-central Africa from where it spread around the world [6,7,8]. After an initial expansion throughout Africa, it reached the Eurasian continent after the last glaciation around 10,000 years ago [9,10], and later moved on to colonize Asia and Europe [11]. An important limiting factor while settling in Europe and at high altitudes in sub-Saharan Africa must have been temperate climates, with their low and varying temperatures.

For *Drosophila*, it is known that the transcriptional output of genes can be affected by temperature [12,13]. The resulting expression plasticity across temperatures might have been detrimental in temperate climates if it shifts the transcriptional output away from the optimum. A high degree of expression plasticity, for instance, could have severe consequences for the intricate interactions of genes involved in development and cell differentiation. The reduction of temperature-induced expression plasticity of functionally important genes, therefore, might have been important while adapting to temperate environments, in order to buffer against fluctuations in temperature and to maintain consistent expression levels across temperatures [14]. Indeed, temperature-induced gene expression plasticity was generally reduced in a temperate compared to a tropical population, both from Australia [12].

The gene vestigial (*vg*), well-known for its wing mutants, is a key player in the development of the *Drosophila* wing. It encodes a transcription factor that plays an essential role in the development and patterning of the wing [15]. Loss of *vg* results in the failure of wings to develop [16] and ectopic expression of *vg* leads to the outgrowth of ectopic wing tissue [17]. Known *vg* mutations display a range of temperature-sensitive expression patterns [18] indicating that DNA sequence changes at the *vg* locus can cause differences in temperature-sensitivity. This makes *vg* an interesting candidate for studying temperature-sensitive gene expression and the potential buffering thereof in flies from temperate climates.

We investigated temperature-sensitive expression of *vg* in natural populations of *D. melanogaster* from six different locations. These included three temperate populations from Europe, one temperate population from a high-altitude location in Africa, and two tropical populations from the ancestral species range. Temperature-induced plasticity of *vg* expression with higher expression at lower temperature appeared to be restricted to certain tissues and/or stages. The degree of expression plasticity differed between the populations with a higher degree in those from hot climates than in those from temperate climates. In addition, *vg* expression was significantly increased across temperatures in cold-temperate flies.

## 2. Materials and Methods

### 2.1. Expression Analysis

Six population samples from Sweden (Umeå), The Netherlands (Leiden), France (Lyon), Rwanda (Gikongoro), Zimbabwe (Lake Kariba), and Zambia (Siavonga) (Table 1) were selected for expression analysis in adult *D. melanogaster*. Tissue-specific expression analysis in third instar larvae was performed using samples of four of the aforementioned populations from Sweden, The Netherlands, Zimbabwe, and Zambia. Each population sample consisted of a pool of eight isofemale lines from the respective population. Flies were reared on a standard cornmeal-molasses medium with a 14/10 h light/dark cycle at 17 °C or 28 °C. Five males and five females were allowed to mate and oviposit for seven or three days at 17 °C and 28 °C, respectively. Flies reared at 17 °C were allowed to oviposit for a longer period since development is slowed at lower temperatures. Mated adult males (aged 4–6 days after eclosion) of the resulting progeny, with one male of each line of the respective population, were pooled for RNA extraction resulting in a pool of eight flies per sample. For tissue-specific expression-analysis, 50 eggs per vial were allowed to hatch and develop into wandering third instar larvae (wL3) from which tissue was sampled. Eight brain and 16 wing imaginal discs of each line were pooled for RNA extraction resulting in a pool of 64 brains and 128 discs per sample, respectively. After dissection, tissues were immediately stored in RNAlater (Qiagen, Hilden, Germany). RNA was extracted using the MasterPure RNA Purification Kit (Epicentre, Madison, WI, USA). RNA purity was assessed via the ratio of absorbances at 260 and 280 nm (A260/A280 > 1.8). It was then reverse transcribed into cDNA using random primers and SuperScript® III Reverse Transcriptase (Invitrogen, Carlsbad, CA, USA). The RT-qPCR reactions were performed with iQ™ SYBR^®^ Green Supermix (BioRad, Hercules, CA, USA) on a CFX96™ real-time PCR cycler (BioRad, Hercules, CA, USA). Primers for target genes and two reference genes for normalization (*RpS20* and *RpL32*) were designed applying the QuantPrime software [19]. Primer sequences are given in Supplementary Table S1. Three biological replicates per population sample, rearing temperature and tissue were run in triplicates. Primer specificity was confirmed by a melting curve analysis. The PCR efficiency was above 95% for all primer pairs. Negative controls included no template controls (NTCs) and no reverse-transcription controls (NRTs) to exclude contamination. Relative expression was calculated using the qBase relative quantification framework [20]. Both reference genes were stably expressed across samples. This was assessed by calculating the coefficient of variation and the M stability parameter according to Hellesmans et al. [20]. Log-transformed normalized relative quantities were subjected to statistical analysis. The effects of rearing temperature and climate were analyzed using a mixed linear model with rearing temperature, climate as well as their interaction as fixed effects factors, and population as a random effects factor. Models were fitted employing REML estimation. Significance of fixed effects was estimated using Type III Wald Chi-square tests. Welch Two Sample t-tests were done for pairwise comparisons. between the different rearing temperatures and population samples. False discovery rate was controlled applying the multiple testing correction method by Benjamini and Hochberg [21].

### 2.2. Sequence Analysis

Sequence data of multiple genomes from the French (96 lines), Rwandan (27 lines), and Zambian (197 lines) populations were derived from the population genomic resource *Drosophila* Genome Nexus (DGN) [22]. The Dutch population used in the expression analysis was not available through the DGN. Another Dutch sample (19 lines) included in the DGN, which was collected only about 60 km away from Leiden in Houten was instead used in the analysis. Genomes from the Swedish population (14 lines) [23] were aligned using the DGN alignment pipeline [22]. *F_ST_* was estimated according to Hudson et al. [24] and assessed per SNP in a 300-kB window surrounding *vg*. SNPs with less than seven called alleles in one or more of the populations were excluded from the analysis. The *vg* gene region was defined as the interval between the two outer insulators [25] flanking the *vg* locus.

## 3. Results

### 3.1. Expression Analysis in Adult D. melanogaster

Male adult flies from different natural populations were reared at two different temperatures (17 °C and 28 °C) to examine whether *vg* is exhibiting temperature-sensitive expression. Population samples were from six locations of different climates (Table 1). Samples from tropical regions included two from the ancestral *D. melanogaster* species range: Zambia and Zimbabwe [8]. The other four samples were derived from populations of temperate regions: three from warm-temperate climates including two European samples (France and The Netherlands) and an African high-elevation population sample from Rwanda, and one from the cold-temperate climate of Sweden.

**Figure 1:**
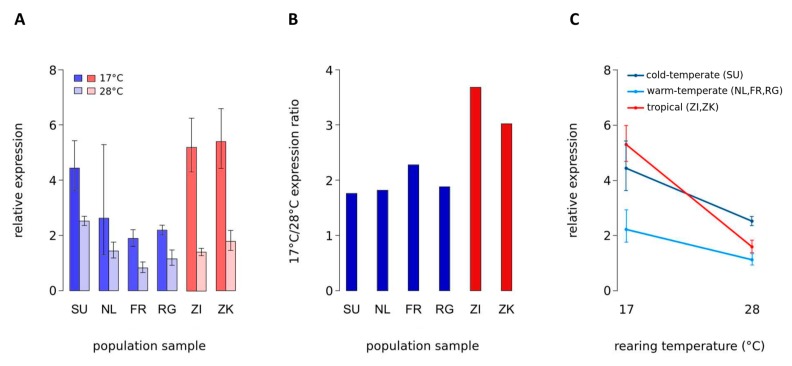
Expression of *vg* in adult *D. melanogaster*. mRNA abundance in adult males was measured via RT-qPCR. (**A**) Expression in population samples from temperate Sweden (SU), Netherlands (NL), France (FR), and Rwanda (RG; high-altitude) (blue bars), as well as in tropical samples from Zambia (ZI) and Zimbabwe (ZK) (red bars). Flies were reared at either 17 °C or 28 °C. Error bars represent the 95% confidence intervals. (**B**) Ratio of mean *vg* expression between rearing temperatures (17 °C to 28 °C) for all six population samples. (**C**) Expression response to rearing temperature in cold-temperate (SU), warm-temperate (NL, FR, RG), and tropical (ZK, ZI) populations. Error bars indicate the 95% confidence intervals.

**Figure 2:**
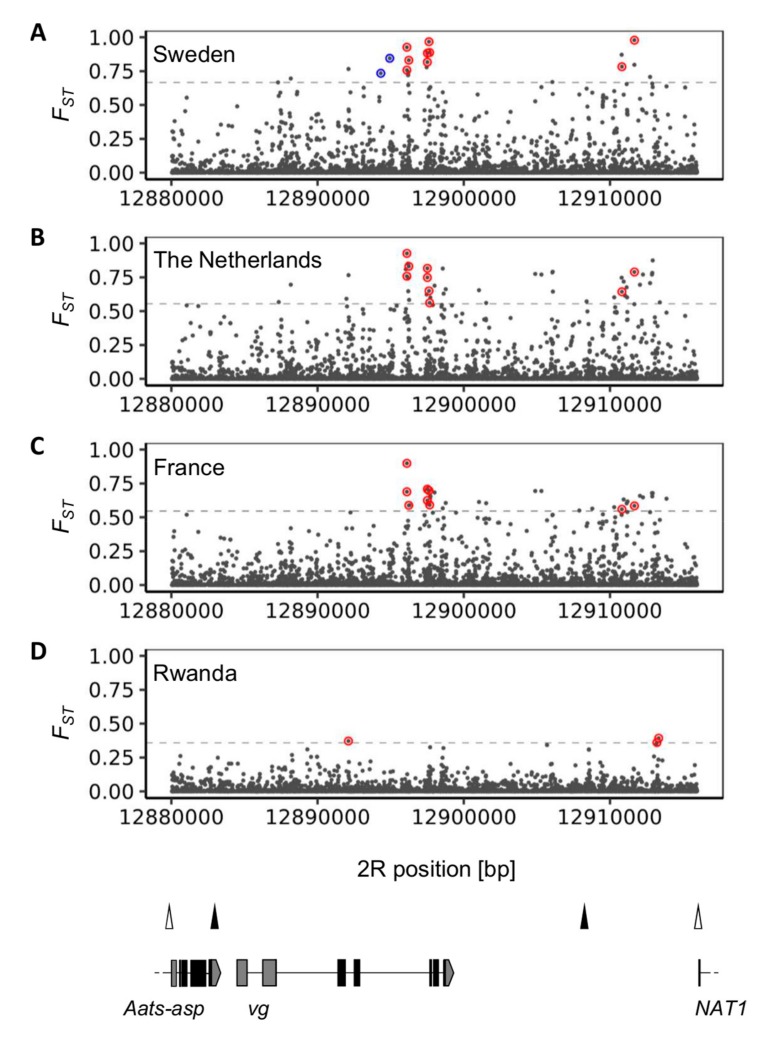
*F_ST_* per SNP across the *vg* gene region. *F_ST_* was estimated between the tropical Zambian population and each of the temperate populations. *F_ST_* between the populations from Sweden (**A**), The Netherlands (**B**), France (**C**) and the tropical, ancestral range population from Zambia. Outlier SNPs shared between all European populations are encircled in red. Dashed lines correspond to the respective 99^th^ quantiles. Outlier SNPs between the Swedish and Zambian population samples and that are also highly differentiated between the Swedish and each of the other three temperate populations (TOP5%) are encircled in blue. (**D**) *F_ST_* between the Rwandan and the Zambian population samples. Outlier SNPs are encircled in red. The dashed line corresponds to the 99.9^th^ quantile. The bottom diagram depicts the *vg* gene region. Coding regions are represented by black boxes, untranslated regions by gray boxes, and introns by black lines. Arrowheads indicate the direction of transcription and positions of insulators are given by triangles.

Expression of *vg* was significantly affected by the temperature at which flies were reared, and by the climate from which they were derived (Figure 1, Table 2). The interaction between rearing temperature and climate of origin was highly significant (Figure 1C, Table 2), suggesting a change in the degree of temperature-induced expression plasticity between populations from different climates. No significant interaction was observed when only the populations from cold- and warm-temperate climates were included in the model. Thus, the amount of change in *vg* expression due to temperature appears to be similar between the populations from cold- and warm-temperate climates. We then fitted a more parsimonious model for the temperate populations without the interaction, which yielded highly significant effects of rearing temperature and climate of origin on the expression of *vg* (Supplementary Table S2). Removing the interaction had no significant effect on the fit of the model (Χ^2^ = 0.2832, *p* = 0.59). Therefore, although the expression response to temperature seems to be similar between flies from cold- and warm-temperate climates, the overall expression of *vg* was higher in cold- than in warm-temperate populations (Figure 1C). Temperature-sensitive gene expression of *vg* due to variation in rearing temperature was observed for all population samples, but not for the control gene *Aats-asp,* which is located adjacent to *vg* (Figure 1, 2,3 ). Expression of *vg* was consistently higher at 17 °C than at 28 °C, and this difference was significant for all samples except for the Dutch one, which exhibited a rather high variation between biological replicates at 17 °C (Figure 1A, Table 3). Interestingly, with a ~2-fold higher expression at 17 °C, the ratio between *vg* expression at 17 °C and at 28 °C was lower in all four temperate population samples than in the tropical samples in which *vg* expression was more than 3-fold higher at 17 °C (Figure 1B, Table 2). Temperature-induced expression plasticity of *vg*, therefore, appears to be buffered in derived temperate genotypes compared to tropical genotypes.

Although the amount of buffering among temperate samples appears to be the same, the mechanisms of how the lowered expression ratio between rearing temperatures comes about seem to be different. For the cold-temperate sample from Sweden, increased *vg* expression relative to the other population samples was observed at 28 °C. The difference was statistically significant for all comparisons between the Swedish and the other samples, except for the one to Zimbabwe, which was of borderline significance (*p* = 0.07) (Supplementary Table S3). In the three warm-temperate population samples, *vg* expression was decreased at 17 °C compared to the other three samples. Statistical significance was found for all comparisons, except for those including the Dutch sample (Figure 1A, Table 3). Again, the reason for this is the high variation between biological samples in the Dutch *vg* expression at 17 °C, which might be eliminated by increasing sample size. At least for the French and Rwandan samples, this decreased *vg* expression is also visible at 28 °C, though to a lower extent than at 17 °C (Figure 1A, Table 2). This could suggest different mechanisms between cold- and warm-temperate populations of how the lowered expression ratio between rearing temperatures arises. More likely, however, given the shared demographic history of European populations, is that, on top of buffering of temperature-sensitive expression in European flies, overall expression of *vg* is additionally elevated in the Swedish population.

### 3.2. Tissue-Specific Expression Analysis in Third Instar Larvae of D. melanogaster

Adult structures are derived from larval structures called imaginal discs. Since the *vg* gene product has its main function in the control of wing formation [15,16,17], the *vg* gene shows an enriched expression in wing imaginal discs [15]. This tissue was, therefore, chosen to further examine *vg* expression under different rearing temperatures. The same was done for larval brains in order to monitor *vg* expression in a tissue in which expression of the gene is known to be low [27]. Gene expression was measured in four samples of the aforementioned populations from Sweden, The Netherlands, Zambia and Zimbabwe (Table 1). As expected, *vg* expression was significantly higher in wing discs than in brains at both rearing temperatures and across all populations (Supplementary Table S4). Temperature-sensitive expression with increased expression levels at lower temperatures, as observed for adults in this study, was neither detected in wing discs nor in brains for *vg* or the control gene Aats-asp (Supplementary Table S5). Decreased expression levels in the Zambian sample at 17 °C relative to 28 °C were the only statistically significant differences observed in *vg* expression (Supplementary Table S5, Supplementary Table S6). Thus, neither temperature-sensitivity in *vg* expression as observed for adults nor a buffering of it in temperate populations appear to play a role in the larval tissues examined here.

### 3.3. Sequence Analysis of Genetic Differentiation at the vg Locus

Both trans-regulatory and *cis*-regulatory changes might be responsible for the expression changes observed in adult flies. Potential *cis*-regulatory changes should be sufficiently differentiated between populations. In order to identify candidates of *cis*-regulatory changes between temperate and tropical populations that might be contributing to the observed expression differences, we estimated *F_ST_* per SNP between each of the four temperate populations and the tropical, ancestral range population from Zambia in a 300-kB window around *vg*. We then looked for outlier SNPs (TOP *F_ST_* values) in the *vg* gene region (see Materials and Methods). No shared outlier SNPs (TOP1% *F_ST_*) were detected when all temperate populations were considered (Figure 2). Given their geographic closeness and their shared demographic history [10,11,23], a common genetic basis for the reduced plasticity in *vg* expression is rather likely in the case of the European temperate populations. Nine outlier SNPs (TOP1% *F_ST_*) were found to be shared between the three European populations (Figure 2; Supplementary Table S7). Since overall expression in the Swedish population appears to be elevated compared to the other temperate populations, we also looked for outlier SNPs (TOP1% *F_ST_*) that are also highly differentiated between the Swedish and each of the other temperate populations (TOP5% *F_ST_*). We observed two such SNPs which are located in the fourth intron of *vg* (Figure 2, Supplementary Table S7). The TOP1% *F_ST_* cutoff value between the two sub-Saharan African populations from Rwanda and Zambia was relatively low (0.19). Thus, we only considered the TOP0.1% *F_ST_* values as possible candidate SNPs for reduced plasticity in *vg* expression in the Rwandan population. Three such SNPs were observed (Figure 2, Supplementary Table S7).

## 4. Discussion

Here we examined the expression response to temperature of the gene *vg* in six natural populations of *D. melanogaster* from different latitudes and altitudes. *vg* is a transcription factor that is known as a wing selector gene due to its essential role in the development and patterning of the *Drosophila* wing. In all four temperate populations, temperature-sensitive expression plasticity was reduced compared to the two tropical populations from the ancestral species range. Temperate populations were derived from a range of different locations including high-latitude Europe and high-altitude Africa. The consistent response to temperature across all temperate populations is consistent with positive selection acting to reduce temperature-sensitivity of *vg* expression in temperate climates.

We observed that reduced *vg* expression at 17 °C compared to the expression level in tropical flies led to a buffering of temperature-induced expression plasticity in the three population samples from warm-temperate climates. In contrast, in the cold-temperate sample from Sweden, increased *vg* expression at 28°C relative to the tropical and the other population samples resulted in the observed buffering effect. Given the shared demographic history of European populations, it seems likely that in addition to buffering temperature-sensitive expression in European flies, overall expression of *vg* is increased in the Swedish population. Other ecological constraints due to the colder climate in Sweden could be a possible explanation for the observed difference. Higher overall *vg* expression, for instance, might have been further beneficial in the colder climate of Sweden.

The observed direction of the expression response to temperature with higher expression at lower temperatures is typical for genes regulated by the Polycomb group (PcG) [28,29,30,31,32]. As for many developmentally important genes, the expression of *vg* is epigenetically controlled by this group of proteins. Interestingly, an earlier study found evidence for selection acting on cis-regulatory sites leading to reduced expression plasticity of another PcG-target gene in temperate flies [33]. The selected sites were highly differentiated between African and European *D. melanogaster* populations and were located in a Polycomb response element (PRE), a cis-regulatory DNA element that recruits PcG proteins to their target genes [34].

Although *vg* plays important roles in the differentiation of adult structures during development [15,16,17,35], little is known about its function in adult flies. Since the main function of *vg* is in wing development [15,16,17], we also looked for temperature-induced expression plasticity and its possible buffering in wing discs of wandering third instar larvae. In this tissue and at this developmental stage, *vg* is in an activated state and highly expressed [15,27]. We chose the larval brain, in which *vg* gene expression is low, as a control tissue [27]. Temperature-sensitive expression as it is often observed for PcG-regulated genes and as we found for adults was not detected in either of the two larval tissues. Possible explanations for this include that selective pressure against such a temperature-induced expression plasticity might be much stronger in larval tissues compared to adult tissues and therefore is not observed in any of the populations. Alternatively, *vg* expression is in itself not affected by temperatures in larval tissues like it is in adult tissues. At least for wing discs, the former explanation might be more likely. Mutations in *vg* introns were found to cause temperature-sensitive expression of the gene in wing imaginal discs, whereas no temperature-sensitivity was observed for wild type discs [18].

Both trans-regulatory and cis-regulatory changes might be responsible for the changes in *vg* expression in adult flies. A range of temperature-sensitive expression patterns of *vg* has been observed in mutant flies carrying mutations at the *vg* locus [18] indicating that DNA sequence changes at the *vg* locus can cause differences in temperature-sensitivity. We assessed genetic differentiation in the *vg* gene region in an attempt to identify potential candidates for cis-regulatory changes responsible for the observed expression differences. Candidate SNPs were located in the introns of *vg* and upstream of *vg* in a region also occupied by the insulators that demarcate the *vg* gene region. As mentioned above, mutations in introns of *vg* can change temperature-sensitivity of *vg* expression [18], whereas insulators are known to play important roles in ensuring PcG-mediated gene repression [36,37,38,39]. One of the Rwandan candidate SNPs was found in a region in the third intron of *vg*, which, if disrupted by a mutation, was shown to result in increased temperature-sensitive expression of *vg* [18]. A common cis-regulatory mechanism between the temperate European and Rwandan populations appears to be less likely, since no shared outlier SNPs were observed. However, whether the detected candidate SNPs are actually involved in changes of *vg* expression observed in this study remains unclear. Trans-regulatory changes also might play a role, as well as sites and indels that were not included in the analysis. Furthermore, elevated genetic differentiation of SNPs might also result from neutral processes such as demographic processes or due to being linked to selected sites not involved in the regulation of *vg* gene expression.

## Figures and Tables

**Table 1:** Population samples.

Population Sample	Latitude	Longitude	Altitude (m)	Mean Annual Temperature (°C)	Minimum Annual Temperature (°C)	Maximum Annual Temperature (°C)	Climate
Umeå, Sweden (SU)	63.83	20.26	12	2.7	−1.6	7.0	cold-temperate
Leiden, The Netherlands (NL)	52.17	4.48	0	9.5	6.4	12.6	warm-temperate
Lyon, France (FR)	45.76	4.84	175	11.6	6.9	16.4	warm-temperate
Gikongoro, Rwanda (RG)	−2.49	28.92	1927	17.6	12.4	23.0	warm-temperate
Lake Kariba, Zimbabwe (ZK)	−16.52	28.80	619	25.5	18.7	31.9	tropical
Siavonga, Zambia (ZI)	−16.54	28.72	530	25.2	18.6	31.9	tropical

Climate data was taken from http://en.climate-data.org/. [26]

**Table 2:** Analysis of variance for effects of rearing temperature and climate of origin on *vg* expression.

	Χ^2^	df	*p*
**Adult**			
Intercept	32.6367	1	<0.001 ***
Rearing temperature	9.9029	1	0.001650 **
Climate of origin	19.7739	2	<0.001 ***
Rearing temperature x climate of origin	12.7246	2	0.001725 **
**wL3 wing imaginal discs**			
Intercept	1.4627	1	0.2265
Rearing temperature	0.0066	1	0.9354
Climate of origin	0.3989	2	0.8192
Rearing temperature x climate of origin	1.4034	2	0.4957
**wL3 brains**			
Intercept	19.8352	1	<0.001 ***
Rearing temperature	2.0164	1	0.1556
Climate of origin	1.6951	2	0.4285
Rearing temperature x climate of origin	1.0706	2	0.5855

Significance of fixed effects was estimated using Type III Wald Chi-square tests. ** *p* < 0.01, *** *p* < 0.001; three climates of origin were considered in the analysis: cold-temperate, warm-temperate, and tropical. Rearing temperature, climate of origin as well as their interaction were treated as fixed effects factors, and population as a random effects factor.

**Table 3:** Fold-changes in gene expression between rearing temperatures in adult *D. melanogaster*.

Gene	Gene Expression Ratio 17/28 °C					
	Sweden (SU)	The Netherlands (NL)	France (FR)	Rwanda (RG)	Zimbabwe (ZK)	Zambia (ZI)
*vg*	1.76 *	1.82	2.28 **	1.88 **	3.68 **	3.02 **
*Aats-asp*	1.15	1.52	0.90	0.92	1.01	1.17

Statistical testing included *t*-tests and correction for multiple testing. * *p* < 0.05, ** *p* < 0.01 (FDR = 0.05).

## Supplementary Materials

Table S1: Primer sequences, Table S2: Analysis of variance for effects of rearing temperature and climate of origin on vg expression including only temperate populations, Table S3: Fold-changes in gene expression between population samples in adult *D. melanogaster*, Table S4: Fold-changes in gene expression at different rearing temperatures between tissues in third instar larvae, Table S5: Fold-changes in tissue-specific gene expression between rearing temperatures in third instar larvae, Table S6: Fold-changes in tissue-specific gene expression between population samples in third instar larvae, Table S7: candidate SNPs.
