# Supplementary material for: Decreased Temperature Sensitivity of Vestigial Gene Expression in Temperate Populations of Drosophila melanogaster
Source: Genes (Basel). 2019 Jun 28;10(7):498. doi: 10.3390/genes10070498 (PMC6679080; doi:10.3390/genes10070498)
Supplement: Supplementary file 1 [file genes-10-00498-s001.pdf]

Supplementary Materials

Table S1. Primer sequences

Gene	Forward primer sequence	Reverse primer sequence
vg	GCGCCCTCAACTACAACAACAAG	GGTGCCATACAAGTCGCTAACCTG
Aats-asp	GCCAAGCACCATGGCATTGATAC	ACATCCATAGCGGAAGGACTCG
RpS20	TTCGCATCACCACCCGTAAGAC	TTGTGGATTCTCATCTGGAAGCG
RpL32	ATCGTGAAGAAGCGCACCAAGC	TTGCGCCATTTGTGCGACAG

Table S2. Analysis of variance for effects of rearing temperature and climate of origin on *vg* expression including only temperate populations

	X^2	df	*P*
Adult – model with interaction:			
(Intercept)	25.5708	1	< 0.001***
Rearing temperature	7.9145	1	0.004904 **
Climate of origin	0.9002	1	0.342728
Rearing temperature x climate of origin	0.2567	1	0.612424
Adult – model without interaction:			
(Intercept)	72.2112	1	< 0.001***
Rearing temperature	44.0250	1	< 0.001***
Climate of origin	8.6939	1	0.003193 **

Significance of fixed effects was estimated using Type III Wald Chi-square tests. **P<0.01, ***P<0.001; Two climates of origin were considered in the analysis: cold-temperate and warm-temperate.

Table S3. Fold-changes in gene expression between population samples in adult *D. melanogaster*

Gene	Gene expression ratio between population samples		
	Population samples	Rearing temperature	
		17°C	28°C
vg	Sweden (SU)/The Netherlands (NL)	1.69	1.75*
	Sweden (SU)/France (FR)	2.35**	3.04**
	Sweden (SU)/Rwanda (RG)	2.03*	2.16**
	Sweden (SU)/Zimbabwe (ZK)	0.82	1.41
	Sweden (SU)/Zambia (ZI)	0.85	1.78***
	The Netherlands (NL)/France (FR)	1.39	1.75*
	The Netherlands (NL)/Rwanda (RG)	1.20	1.24
	The Netherlands (NL)/Zimbabwe (ZK)	0.49	0.81
	The Netherlands (NL)/Zambia (ZI)	0.50	1.02
	France (FR)/Rwanda (RG)	0.86	0.71
	France (FR)/Zimbabwe (ZK)	0.35**	0.65**
	France (FR)/Zambia (ZI)	0.36**	0.59*
	Rwanda (RG)/Zimbabwe (ZK)	0.41**	0.65**
	Rwanda (RG)/Zambia (ZI)	0.42**	0.82
	Zimbabwe (ZK)/Zambia (ZI)	1.04	0.79
Aats-asp	Sweden (SU)/The Netherlands (NL)	0.69*	0.92
	Sweden (SU)/France (FR)	0.96	0.76
	Sweden (SU)/Rwanda (RG)	0.88	0.70
	Sweden (SU)/Zimbabwe (ZK)	0.90	0.91
	Sweden (SU)/Zambia (ZI)	0.87	0.76
	The Netherlands (NL)/France (FR)	1.39	0.83
	The Netherlands (NL)/Rwanda (RG)	1.26	0.77
	The Netherlands (NL)/Zimbabwe (ZK)	1.29	0.99
	The Netherlands (NL)/Zambia (ZI)	1.25	0.83
	France (FR)/Rwanda (RG)	0.91	0.93
	France (FR)/Zimbabwe (ZK)	0.93	1.20
	France (FR)/Zambia (ZI)	0.90	1.00
	Rwanda (RG)/Zimbabwe (ZK)	1.02	1.29
	Rwanda (RG)/Zambia (ZI)	0.99	1.08
	Zimbabwe (ZK)/Zambia (ZI)	1.03	1.20

Statistical testing included t-tests and correction for multiple testing. $^{*}P<0.05$, $^{**}P<0.01$, $^{***}P<0.001$ (FDR=0.05).

Table S4. Fold-changes in gene expression at different rearing temperatures between tissues in third instar larvae

Gene	Rearing temperature	Gene expression ratio wing disc/brain			
		Sweden (SU)	The Netherlands (NL)	Zimbabwe (ZK)	Zambia (ZI)
vg	17°C	11.77***	10.53**	13.19***	14.64**
	28°C	8.45***	12.71**	8.59***	12.35***
Aats-asp	17°C	1.10	0.99	1.10	1.32
	28°C	1.04	1.13	1.20	1.23

Statistical testing included t-tests and correction for multiple testing. **P<0.01, ***P<0.001 (FDR=0.05).

Table S5. Fold-changes in tissue-specific gene expression between rearing temperatures in third instar larvae

Gene	Tissue	Gene expression ratio 17°C/28°C			
		Sweden (SU)	The Netherlands (NL)	Zimbabwe (ZK)	Zambia (ZI)
vg	Wing disc	1.02	0.71	1.29	0.59*
	Brain	0.73	0.85	0.84	0.50*
Aats-asp	Wing disc	0.95	0.87	0.99	0.88
	Brain	0.90	0.99	1.07	0.81

Statistical testing included t-tests and correction for multiple testing. *P<0.05 (FDR=0.05).

Table S6. Fold-changes in tissue-specific gene expression between population samples in third instar larvae

Gene	Tissue	Gene expression ratio between population samples		
		Population samples	Rearing temperature	
			17°C	28°C
vg	Wing disc	Sweden (SU)/The Netherlands (NL)	0.86	0.60
		Sweden (SU)/Zimbabwe (ZK)	0.75	0.74
		Sweden (SU)/Zambia (ZI)	1.35	0.78
		The Netherlands (NL)/Zimbabwe (ZK)	0.87	1.59
		The Netherlands (NL)/Zambia (ZI)	1.56	1.30
		Zimbabwe (ZK)/Zambia (ZI)	1.79*	0.82
	Brain	Sweden (SU)/The Netherlands (NL)	0.77	1.14
		Sweden (SU)/Zimbabwe (ZK)	0.84	0.90
		Sweden (SU)/Zambia (ZI)	1.67	0.97
		The Netherlands (NL)/Zimbabwe (ZK)	1.09	1.07
		The Netherlands (NL)/Zambia (ZI)	2.17*	1.27
		Zimbabwe (ZK)/Zambia (ZI)	1.99	1.18
Aats-asp	Wing disc	Sweden (SU)/The Netherlands (NL)	0.94	0.86
		Sweden (SU)/Zimbabwe (ZK)	0.84	0.87
		Sweden (SU)/Zambia (ZI)	1.06	0.97
		The Netherlands (NL)/Zimbabwe (ZK)	0.90	1.01
		The Netherlands (NL)/Zambia (ZI)	1.13	1.13
		Zimbabwe (ZK)/Zambia (ZI)	1.26	1.12
	Brain	Sweden (SU)/The Netherlands (NL)	0.85	0.93
		Sweden (SU)/Zimbabwe (ZK)	0.84	1.00
		Sweden (SU)/Zambia (ZI)	1.27	1.14
		The Netherlands (NL)/Zimbabwe (ZK)	1.00	1.08
		The Netherlands (NL)/Zambia (ZI)	1.50	1.23
		Zimbabwe (ZK)/Zambia (ZI)	1.51*	1.15

Statistical testing included t-tests and correction for multiple testing. *P<0.05 (FDR=0.05).

Table S7. Candidate SNPs

Populations	Genomic position
Sweden	2R: 12894323
	2R: 12894933
Sweden - The Netherlands - France	2R:12896107
	2R:12896108
	2R:12896238
	2R:12897506
	2R:12897508
	2R:12897618
	2R:12897665
	2R:12910816
	2R:12911672
Rwanda	2R:12892106
	2R:12913210
	2R:12913330

Genomic positions are given according to FlyBase release 6 [1].

References

1. Thurmond, J.; Goodman, J.L.; Strelets, V.B.; Attrill, H.; Gramates, L.S.; Marygold, S.J.; Matthews, B.B.; Millburn, G.; Antonazzo, G.; Trovisco, V.; et al. FlyBase 2.0: the next generation. Nucleic Acids Res. 2019, 47, D759–D765.
